# An Enhanced Electrochemiluminescence Immunoassay Platform via Optimized Magnetic Bead Uniformity for Reliable Thyroid-Stimulating Hormone Monitoring

**DOI:** 10.3390/bioengineering13030333

**Published:** 2026-03-13

**Authors:** Hengbo Lei, Xinyu Huang, Xiang Cao, Yuguo Tang, Yang Ge

**Affiliations:** 1 School of Biomedical Engineering (Suzhou), Division of Life Sciences and Medicine, University of Science and Technology of China, Hefei 230026, China; leihb@sibet.ac.cn; 2 Suzhou Institute of Biomedical Engineering and Technology, Chinese Academy of Sciences, Suzhou 215163, China; 3 Shenyang Guoke Bright Medical Technology Co., Ltd., Shenyang 110170, China

**Keywords:** electrochemical luminescence system, magnetic bead arrangement, image processing, TSH detection

## Abstract

Electrochemiluminescence immunoassay (ECLIA) is widely used in clinical diagnostics owing to its high sensitivity, broad dynamic range, and excellent analytical stability. However, the influence of magnetic bead deposition behavior on electrochemiluminescence (ECL) signal performance remains insufficiently characterized. In this study, a quantitative evaluation method for magnetic bead distribution uniformity on the electrode surface was established and applied to optimize fluidic parameters in an ECLIA measurement system. By combining microscopic imaging with image analysis, magnetic bead spreading behavior under different flow conditions was systematically characterized and correlated with luminescence signal intensity. Optimization of the flow rate (18.46 µL·s^−1^) improved bead distribution uniformity and resulted in a 26.32% increase in luminescence intensity without altering bead coverage or assay chemistry. The optimized system was further validated using thyroid-stimulating hormone (TSH) detection, showing a linear response over 0.016–120 µIU·mL^−1^ (R^2^ > 0.996) and high consistency with a commercial analyzer (R^2^ = 0.998) from Roche. These results demonstrate that quantitative control of magnetic bead distribution provides an effective strategy for improving ECLIA performance and offers a general optimization framework for bead-based electrochemiluminescence systems.

## 1. Introduction

Electrochemiluminescence (ECL), also referred to as electrogenerated chemiluminescence, is a phenomenon in which luminophores emit light through high-energy electron transfer during an electrochemical reaction on the electrode surface [1]. As ECL depends on external optical excitation, it features merits such as low detection limit, wide linear range, fast response kinetics, and excellent stability and has attracted considerable attention in biological sensing and clinical diagnostics [2]. Among various ECL systems, the tris(2,2′-bipyridyl)ruthenium(II) (Ru(bpy)_3_^2+^)/tripropylamine (TPrA) system remains one of the most established configurations [3,4]. Modern electrochemiluminescence immunoassay (ECLIA) platforms commonly employ streptavidin-coated magnetic beads as solid-phase carriers, enabling automated enrichment, separation, and signal amplification [5]. These systems integrate key assay steps including reagent dispensing, dilution, incubation, washing, and signal acquisition and have caught widespread interest in clinical and biochemical analysis [6,7]. The foregoing platform has been successfully commercialized for quantitative detection of various biomarkers [8]. The core of such platform is an ECL detection module, whose key feature is the directional immobilization of magnetic beads, allowing precise control over the electrochemical reaction. Thus, stable and reproducible ECL signals can be generated [9]. Typically, a detection module relies on the high-affinity biotin-streptavidin interaction. Streptavidin-coated magnetic beads serve as solid-phase carriers for directional immobilization of biotinylated capture antibodies [10]. Target analytes, such as antigens in the sample, are then captured via a sandwich immunoassay format. Subsequently, detection antibodies labeled with ECL tags bind to the formed antigen-antibody complex. This enables specific detection, and a quantifiable luminescent signal upon electrochemical stimulation is generated [11,12]. As a result, the system supports the detection of a wide spectrum of pathological markers, including those associated with cardiovascular diseases, infectious diseases, thyroid disorders, cancers, as well as bacterial and viral pathogens [13].

However, as a surface-confined technique, ECL can effectively generate signals only when immunocomplexes labeled with luminophores are in close proximity to the electrode surface, due to the extremely short lifetime of coreactant radicals [14,15]. Previous studies have shown that the strongest ECL emission originates within a microregion only a few micrometers from the electrode surface [16]. Therefore, the spatial distribution of magnetic beads on the working electrode plays a critical role in determining signal intensity and detection reliability. Enhanced distribution uniformity increases the proximity of the luminescent species to the electrode surface, leading to improved ECL efficiency [17]. Signal quenching or false-negative outcomes may arise from abnormal bead distribution on the electrode surface, posing a substantial risk to detection reliability. Non-uniform bead deposition may lead to inefficient electron transfer, signal attenuation, or false-negative results. Despite its importance, magnetic bead handling in most commercial systems is treated as an empirical or system-dependent process, and quantitative evaluation of bead distribution and its relationship with detection performance remains limited. Recent advances in ECL microscopy have enabled visualization of electrochemical reaction zones and spatial signal distribution [18,19,20]. The electrochemical excitation process in an ECL reaction does not require the incident excitation light, which is necessary in the fluorescence techniques. Therefore, ECL exhibits extremely low background noise and a high signal-to-noise ratio, leading to significant advantages for analytical detection, quantitative evaluation, and microscopy imaging applications [21]. This character further expands the application potential of ECL in microfluidic detection and point-of-care testing (POCT) [22,23]. Nevertheless, these studies primarily focus on reaction mechanisms rather than systematic analysis of magnetic bead deposition behavior or its direct impact on analytical performance. A quantitative framework linking bead distribution uniformity with ECL signal enhancement is still lacking.

To address this limitation, this work develops an integrated ECLIA platform to quantitatively characterize and optimize magnetic bead deposition behavior. Unlike previous studies that primarily visualized ECL reaction mechanisms or treated magnetic bead handling as an empirical black-box process, this study establishes the first quantitative framework that directly links magnetic bead distribution uniformity to measurable signal enhancement. Image acquisition and processing methods were employed to systematically quantify bead coverage and its standard deviation across the electrode surface, transforming bead deposition from an uncontrolled variable into a tunable performance parameter. By systematically investigating fluidic parameters including flow velocity and measurement cell configuration, the relationship between bead distribution and signal performance was clarified. Rather than introducing new assay chemistry, this study demonstrates that improving distribution uniformity alone leads to a 26.32% increase in luminescence intensity without altering bead coverage or assay composition. Thyroid-stimulating hormone (TSH) was selected as a model analyte to evaluate analytical performance, including linearity, repeatability, and agreement with a commercial analyzer. The proposed methodology offers a practical, universally applicable strategy for optimizing magnetic bead distribution and improving detection reliability in bead-based ECL systems.

## 2. Materials and Methods

### 2.1. Materials

The high-concentration TSH standard was obtained from the National Institute for Biological Standards and Control. The magnetic bead suspension, TSH immunoassay kit, the tripropylamine buffer solution and the cleaning solution were acquired from Roche Diagnostics (Mannheim, Germany). A TSH immunoassay kit includes three main reagents: streptavidin magnetic beads (0.72 mg∙mL^−1^), Biotin-conjugated anti-TSH antibody (R1), and Ru(bpy)_3_^2+^-labeled-anti-TSH antibody (R2). The photomultiplier tube (PMT) was sourced from Hamamatsu Photonics (model: R1617, Iwata-City, Shizuoka, Japan). In the experiment, the original Roche magnetic bead suspension was diluted with buffer solution, and the concentration of magnetic beads was 0.18 mg·mL^−1^.

### 2.2. Principle of ECL Detection System

**Figure 1:**
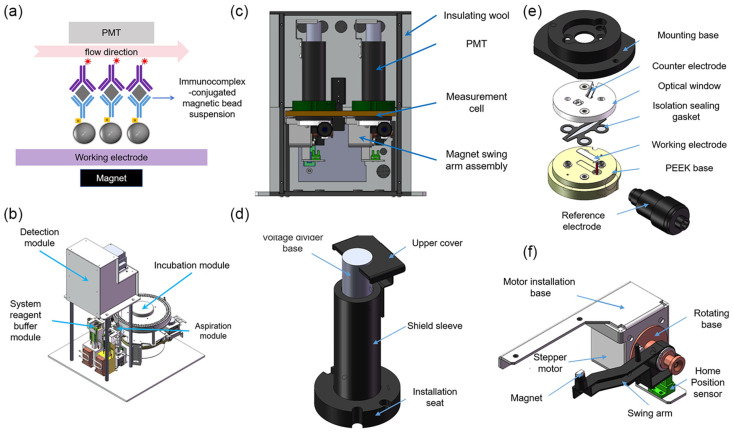
(**a**) Principle of the ECL detection system. The inverted purple Y-shaped structure represents R2, with the asterisk indicating the Ru(bpy)_3_^2+^ label. The gray diamond represents the TSH antigen. The upright blue Y-shaped structure is R1, and the gray sphere represents the streptavidin-coated magnetic bead. Together, these components form the immunocomplex-conjugated magnetic suspension. (**b**) Components of the ECLIA analytical system. (**c**) Overview of the detection module. (**d**) Measurement cell. (**e**) PMT assembly. (**f**) Magnet swing arm assembly.

The working principle of the system is illustrated in Figure 1a [7]. Magnetic beads conjugated with immune complexes are transported into the measurement cell via fluid flow. Under the combined influence of a magnetic field and hydrodynamic forces, the beads are immobilized on the working electrode surface, while unbound interfering substances are removed through washing steps. Subsequently, a potential is applied through a three-electrode electrochemical system to initiate the ECL reaction, resulting in photon emission. The emitted light is detected in real time by a PMT and converted into an electrical signal for data acquisition. Upon completion of the detection cycle, the measurement cell undergoes a regeneration process involving rinsing and electrode activation, restoring the system to its initial state for subsequent assays.

### 2.3. Design of the ECLIA Analytical System

As illustrated in Figure 1b, the entire analytical apparatus is mainly composed of a detection module, an aspiration module, a system reagent buffer module, an incubation module, and the associated fluidic pathways.

The aspiration module consists of a rotational mechanism, a vertical lifting mechanism, and a sampling needle fluidic pathway. According to the requirements of different reaction stages in the ECL detection workflow, the module sequentially performs the quantitative aspiration and transfer of tripropylamine buffer, cleaning solution, and the immune complex-magnetic bead suspension. The system reagent buffer module is designed with an independent dual-cup configuration to separately contain the tripropylamine buffer and cleaning solution, and it integrates a cleaning station for rinsing the outer surface of the sampling needle, thereby effectively preventing cross-contamination. A Peltier-based temperature control system is incorporated into this module, enabling the reagent temperature to be stably maintained at 28 ± 0.3 °C. The incubation module is used for incubating immune complexes and employs a high-precision circumferential heating belt for temperature regulation, providing a constant incubation environment of 37 ± 0.3 °C. This design ensures that immunoreactions proceed under stable and well-controlled thermal conditions, thereby enhancing reaction efficiency as well as the consistency and reliability of the analytical results.

As the core component of the system, the detection module consists of two main parts: a hardware unit and a signal processing unit. As shown in Figure 1c, the hardware of the detection module primarily comprises a measurement cell, a PMT, a magnet driving assembly, and a temperature control system. The detailed structure of the measurement cell is illustrated in Figure 1e. Its main components include a mounting base, a working electrode, a counter electrode, a reference electrode, an optical window, an isolating sealing gasket, and a polyetheretherketone (PEEK) base. The mounting base is fabricated from 6061 aluminum alloy with a blackened surface treatment and serves for overall fixation and mechanical support. A recessed cavity is machined on the upper surface of the base to accommodate the PMT, which is precisely aligned with the central optical aperture of the measurement cell. The optical window is made of high-transmittance acrylic (optical transmittance > 95%), ensuring efficient transmission of ECL photons. The working electrode is a platinum plate (20 × 5 × 0.2 mm) fixed within a recessed groove of a PEEK base. The counter electrode consists of a bent platinum wire (31.3 × 0.5 × 0.3 mm) and is mounted on the inner side of the optical window. An Ag/AgCl electrode is employed as the reference electrode and installed at a designated position on the base. The isolation sealing gasket is made of fluororubber, and its internal diamond-shaped cavity forms the fluidic channel. Two φ1 mm fluidic ports are machined in the base and serve as the inlet and outlet, respectively.

The PMT (Figure 1d) is vertically mounted above the measurement cell, with its housing enclosed in permalloy to effectively shield against magnetic interference. A magnet swing-arm assembly is installed beneath the measurement cell (Figure 1f) to control the distance between the permanent magnet and the cell, thereby enabling controlled adsorption and release of magnetic beads. This assembly consists of a motor mounting plate, a stepper motor, a copper rotating base, a nylon swing arm, and an N42 neodymium–iron–boron permanent magnet. The magnet is fixed at the end of the swing arm, with its magnetic flux lines oriented parallel to, but opposite in direction to, the fluid flow.

The temperature control system comprises a Peltier module integrated at the rear side of the aluminum base and an external thermal insulation layer, allowing the internal temperature of the measurement cell to be stably maintained at 28 ± 0.3 °C. In addition, black rubber sealing gaskets are applied at the joints of the aluminum components to effectively block ambient light interference, thereby ensuring a low-noise optical environment for sensitive ECL detection.

**Figure 2:**
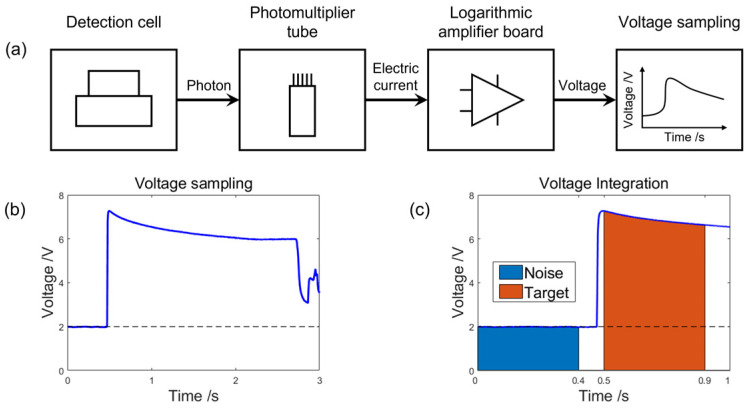
(**a**) Flowchart of signal processing. (**b**) V-t response curve during the reaction process. (**c**) Integration region for signal processing.

Figure 2 shows the system’s signal processing unit, which consists of a logarithmic amplifier board, a data acquisition module, and a data processing module. When photons are emitted from an electrochemical reaction in the measurement cell, they are first detected by a PMT and converted into a weak current signal. This current signal is then sent to the logarithmic amplifier board, where it is amplified and converted from current to voltage. The resulting voltage signal is recorded in real time by the data acquisition module and output as the raw voltage-time (V-t) response curve.

To ensure accurate quantification of the luminescence signal, the data processing algorithm was optimized in this study. The procedure includes the following steps: First, the acquired V-t curve is subjected to an inverse-logarithmic transformation to reconstruct the original current-time (I-t) curve. Next, the amount of charge corresponding to the photons received by the PMT can be calculated as follows:

(1) $$Q_{photon}=S_{target}-S_{noise}$$

Figure 2c shows that the parameter *S*_noise_ indicates the background noise signal, and it can be calculated as the integration of the voltage over the 0.4 s before the electrochemical reaction occurs. The integration time is determined to be 0.4 s to ensure optimal linearity of the integration result. In contrast, *S*_target_ is composed of both the luminescent signals and the background noise signal, which can be obtained by integrating the voltage over the 0.4 s immediately following the reaction onset. Since the background noise is independent of the incident excitation light of the electrochemical reaction, the background noise can be regarded as invariant during the whole reaction. Consequently, the luminescent signal can be obtained by subtracting *S*_noise_ from *S*_target_. The algorithm shown in Figure 2 effectively suppresses background noise, thereby enhancing both the accuracy and repeatability of the detection.

### 2.4. Quantitative Analysis of Magnetic Bead Dispersion

To quantitatively evaluate the distribution of magnetic beads, the following experimental procedure was designed. To begin with, the key fluid parameters, including the flow velocity and the aspiration volume, were adjusted by regulating the operating parameters of the aspiration pump’s stepper motor. Afterwards, an automated sampling needle was used to aspirate the immunocomplex-conjugated magnetic beads suspension (0.18 mg·mL^−1^) into the measurement cell. Moreover, beads adhered to the working electrode surface due to the magnetic field. Meanwhile, microscopic images were acquired to calculate the coverage and uniformity of magnetic beads on the electrode surface.

**Figure 3:**
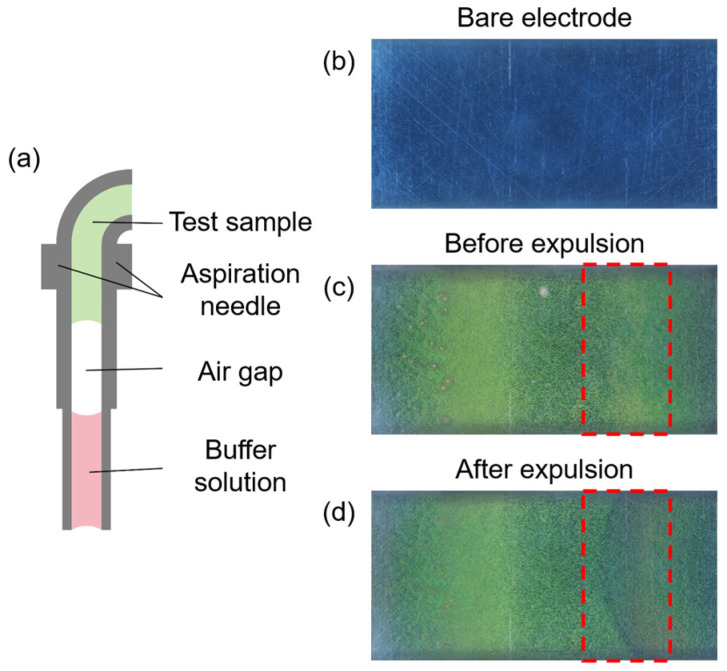
(**a**) Schematic diagram of the air gap within the syringe needle. (**b**) The bare working electrode in the microchannel. (**c**) Distribution of magnetic beads on the working electrode surface before expulsion. (**d**) Distribution of magnetic beads on the working electrode surface after expulsion. The red dashed boxes in figures (**c**,**d**) indicate the crescent shaped pattern formed by the detachment of magnetic beads by air gaps.

During the automated process, cross-contamination can occur when different liquids are aspirated constantly into the fluid path, leading to the inaccuracy in and low reproducibility of assay results. To address this, as shown in Figure 3a, an air gap is aspirated into the fluid system between two different liquids in order to prevent direct contact between liquids and to preserve assay accuracy. As shown in Figure 3c,d, air gaps may peel off magnetic beads already attached to the electrode, particularly in the areas marked by the red dashed boxes in the two images. Due to the surface tension of the liquid and bubble-interface shear effects, smaller air gaps moved faster than expected. This will strip off the magnetic beads already deposited on the working electrode surface. For air gaps with excessive volume, disturbances at the gas-liquid interface may also affect the distribution of magnetic beads. Such phenomena result in waste of magnetic beads, decreased coverage, weakened uniformity, and even the signal instability. Therefore, the air gap parameters must be optimized to balance the minimized disorganization of bead distribution against the reliable separation.

To investigate the influence of flow velocity on the bead distribution, we quantitatively characterized the magnetic bead distribution under a range of flow velocities. Based on the working characteristics of the stepper motor of the aspiration pump, the flow velocity was adjusted by regulating the maximum flow rate and acceleration time (defined as the time it takes to accelerate from initial speed to maximum speed), while the total aspiration volume, initial speed, and the acceleration was held constant. In the experiment, images of the magnetic bead distribution in the measurement cell were captured and processed using a MATLAB script (R2020a, 9.8.0.1323502), whose flowchart can be found in Appendix A. The coverage and uniformity of magnetic beads were calculated by a series of steps, such as RGB channel separation, grayscale conversion, and binarization.

#### 2.4.1. Image Acquisition System Setup

**Figure 4:**
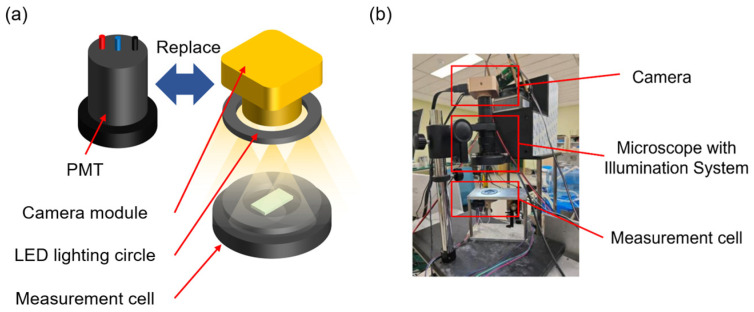
Basic components of the image acquisition system. (**a**) Schematic diagram of using image acquisition components instead of PMT. (**b**) Actual image acquisition system.

In order to acquire the image of the beads adhered to the working electrode surface, an image acquisition system was built and replaced the detection module, which can be seen in Figure 4. The high-resolution camera has 2048 × 2048 pixels and a maximum magnification of 40×. To eliminate the influence of ambient light on image acquisition, images of the magnetic beads inside the microchannel were captured in a darkroom. Illumination was provided exclusively by LED lighting circle surrounding the camera lens, ensuring consistent lighting conditions across all experiments.

#### 2.4.2. Coverage and Uniformity Analysis of Magnetic Beads

**Figure 5:**
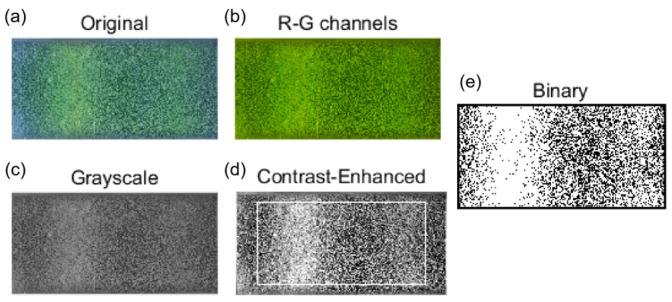
(**a**) A raw image showing magnetic beads distribution in the microchannel of the measurement cell. (**b**) The image obtained by extracting and overlaying the R-G channels. (**c**) Grayscale image. (**d**) Result after enhancing the contrast of the grayscale image (the white square indicates the calculation region). (**e**) Binary image of the selected region, where white represents areas covered by magnetic beads.

In order to quantitatively analyze the coverage and uniformity of magnetic beads, it is necessary to process the raw images captured. The specific flowchart can be found in Appendix A. As shown in Figure 5a, the magnetic beads appear yellow–green in color, and the exposed working electrode appears dark gray. Due to the beads’ low blue-channel intensity, which reduces their contrast against platinum electrodes, the red and green channels were extracted and combined to produce Figure 5b. This image was then converted to grayscale (Figure 5c) and contrast-enhanced, yielding Figure 5d with improved bead-substrate differentiation for further analysis. Coverage was calculated within a rectangular region extracted from Figure 5d. By randomly sampling a large number of grayscale images inside the microchannel and labeling them as belonging to magnetic beads or exposed working electrode surfaces, the grayscale ranges of magnetic beads and electrodes were determined, and the threshold for distinguishing the two was determined. In the binarized representation (Figure 5e), white pixels correspond to beads and black pixels to the working electrode. With *S*_mb_ as the white-pixel count and *S*_base_ as the total pixel count of the region, coverage *C* is defined as follows:

(2) $$C=\frac{S_{mb}}{S_{base}}\times 100\%$$

To assess uniformity, the microchannel was partitioned into nine equally sized rectangular sub-areas arranged in a 3 × 3 grid. The standard deviation (SD) of the coverage among these sub-areas served as the uniformity metric, where a smaller SD corresponds to higher distribution uniformity.

**Figure 6:**
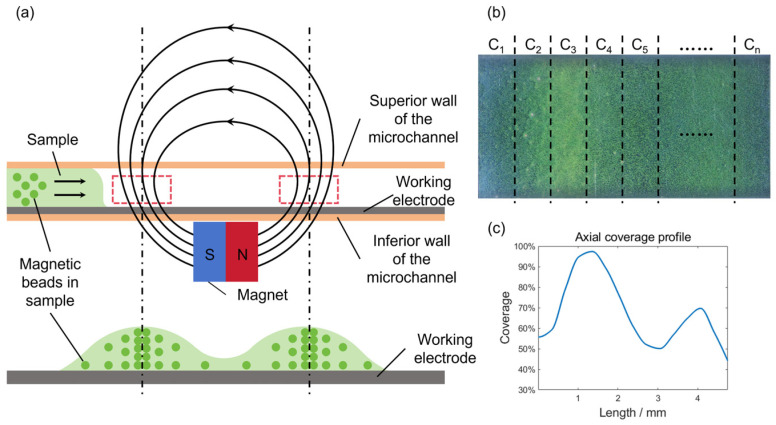
(**a**) Schematic of the flow channel and magnetic bead distribution. The red dashed box indicates the region of stronger magnetic field. (**b**) Schematic illustrating the segmentation of the flow channel for axial coverage calculation. (**c**) Axial coverage profile calculated along the central axis of the flow channel.

As depicted in Figure 5a, the magnetic beads adhering to the working electrode surface formed two clear stripes. This striped pattern results from the magnetic field configuration established by an underlying magnet. The schematic in Figure 6a highlights (with red dashed boxes) two areas of enhanced field strength, where the intensified magnetic force concentrates bead deposition, thereby generating the stripes. To better characterize the magnetic bead distribution, the axial coverage profile is obtained. As shown in Figure 6b, the measurement cell is divided into multiple sub-regions. The coverage of magnetic beads in each subregion is denoted by *C*_n_ and is calculated separately. The distance from the subregion to the inlet of the flow channel is taken as the horizontal axis, and the coverage is taken as the vertical axis. The curve connecting each point is the axial coverage profile, which can be seen in Figure 6c. According to the curve, the magnetic bead coverage shows a saddle-shaped distribution along the axis of the microchannel, with higher density at both ends and lower density in the middle. When magnetic beads flow through the microchannel, they flow past the first location with a stronger magnetic field and are initially captured by the strong magnetic region near the entrance (the red dashed box on the left side of Figure 6a), where more magnetic beads accumulate. The remaining magnetic beads are then captured by the second strong magnetic region further downstream. Thus, the first peak is higher than the second one.

### 2.5. Experiment on the Detection of TSH

The sample processing procedure consists of the following steps: First, 30 μL of the test sample is combined sequentially with 40 μL of reagent R1 and 40 μL of reagent R2 to form an immunocomplex. The mixture is then incubated at 37 °C for 9 min in a constant-temperature shaker. Subsequently, 30 μL of streptavidin-labeled magnetic beads are added, and the mixture is incubated for a specified period. After incubation, the reaction solution is automatically transferred to the measurement chamber using an autosampler for chemiluminescence detection.

To establish the linear range of the detection system, a high-concentration TSH standard was serially diluted in phosphate-buffered saline (PBS, pH 7.2) to generate a series of concentrations (0.016, 0.02, 0.08, 0.2, 0.8, 2, 8, 20, 60, and 120 μIU·mL^−1^). Each diluted sample was processed as aforementioned, and the ECL signal was recorded. Subsequently, linear regression analysis was performed. Here, the independent variable is the concentration of the TSH sample, and the dependent variable is the signal intensity. To ensure the reliability of the system, samples were tested repeatedly (10 times) within the linear range, and the precision was assessed by calculating the coefficient of variation (CV).

To validate the accuracy of the self-developed detection platform, a method-comparison study was conducted using the widely adopted Roche Cobas e411 ECL analyzer as the reference system. The same set of ten samples spanning the concentration range used for linearity assessment was analyzed in parallel on the Roche Cobas e411 instrument. Correlation analysis was performed between the results from both platforms to evaluate their agreement.

## 3. Results and Discussions

### 3.1. Optimization of Fluid Parameters

As mentioned earlier, the distribution of the magnetic beads on the working electrode surface exerts a significant impact on the accuracy of the detection. In this section, our discussion focuses on the experimental parameters that were found to substantially affect the distribution of magnetic beads, including the volume of the air gap, and the flow velocity in the fluidic circuit.

#### 3.1.1. Impact of Air Gap on Magnetic Bead Distribution

As shown in Figure 3, a segment of air is aspirated into the tubing to separate the buffer solution and the immunocomplex-conjugated magnetic beads suspension. After the magnetic beads are attached to the working electrode surface (the bare working electrode can be seen in Figure 3b), an air gap with improper parameters can carry away a portion of the beads, which can be seen in Figure 3c,d. Before the air gap is expulsed, Figure 3c demonstrates the distribution of magnetic beads on the working electrode surface, where bead aggregation forms two clear striped patterns. Figure 3d shows the defect caused by the removal of magnetic beads via the air gap. Such phenomenon leads to decreased coverage and weakened uniformity. To address this issue, we conducted experiments within the achievable air gap volume range based on the detection rate requirements of the detection unit. The air gap volume that would hardly peel off magnetic beads from the electrode surface was selected. In the following experiments, the effects of air gap volumes ranging from 8.75 to 17.5 μL on the distribution of magnetic beads on the working electrode surface were investigated. Images collected both before and after the expulsion of the air gap were provided in Appendix B. Given this, changes in axial coverage profiles varying the volume of air gaps were analyzed.

**Figure 7:**
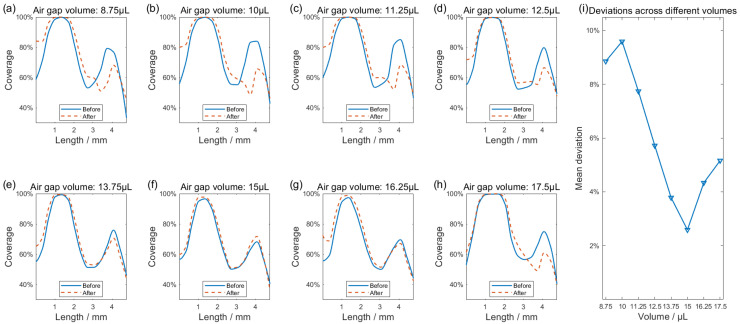
(**a**–**h**) Axial coverage profiles of magnetic beads before and after air gap expulsion, with the isolated air column volume increasing from 8.75 µL to 17.5 µL. (**i**) Dependence of the mean deviation on air gap volume, compared pre- and post-expulsion.

Impact of air gap volume variations on the distribution of magnetic beads on the working electrode surface can be seen in Figure 7. Through blue curves in Figure 7a–g, before the air gaps were expulsed, the magnetic bead coverage profile displayed a saddle-shaped distribution, characterized by a second aggregation peak of reduced height compared to the first. It can be seen that the volume of air gaps predominantly affected the magnetic beads at the second aggregation peak, whereas the alignment between the blue curve and red dashed line remained strong near the first peak independent of the air volume. As Figure 7a indicates, the small-volume air gap carried away some of the magnetic beads already attached to the working electrode surface, resulting in the red dashed curve being significantly lower than the blue curve at the position of the second aggregation peak. The difference between the red dashed curve and the blue curve progressively decreases with increasing air gap volume. A minimal influence of the air gap is observed at 15 μL (Figure 7f), where the two curves essentially merge. Any further increase in air volume, conversely, causes the curves to diverge again. To better characterize the deviation of the post-expulsion red dashed curve relative to the blue reference, the mean coverage difference was calculated in the sub-regions defined in Figure 6b before versus after air gap expulsion. As Figure 7i illustrates, the deviation is lowest at 15 μL, reflecting the strongest agreement between the curves and the least effect of the air gap expulsion on the distribution of the beads. Based on these findings, a volume of 15 µL was selected for the air gap in subsequent experiments while keeping its influence on bead distribution to a minimum.

#### 3.1.2. Impact of Flow Velocity on Magnetic Bead Distribution

**Figure 8:**
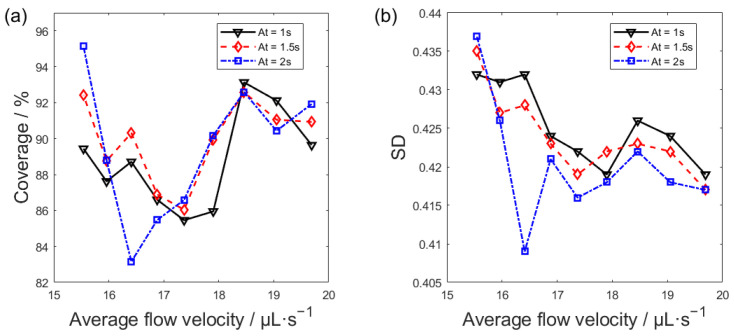
(**a**) Coverage versus flow velocity for magnetic beads. (**b**) Standard deviation versus flow velocity for magnetic beads.

Flow velocity exerts a pronounced effect on the distribution of the immunocomplex-bead suspension in the microchannel. To address this, the average flow velocity and acceleration time were systematically optimized in this study. Images of magnetic bead dispersion in the central channel region under various flow velocities were binarized, with the corresponding bead coverage and its standard deviation calculated, and detailed results can be found in Appendix C. Figure 8 plots the magnetic bead coverage on the working electrode surface and its standard deviation against the average flow velocity in the fluidic system. As presented in Figure 8a, over the tested flow velocity range of about 15–20 μL·s^−1^, with increasing flow velocity, the magnetic bead coverage exhibits a trend of initial decline, subsequent rise, and final reduction. Figure 8b indicates that the increasing flow velocity generally leads to a reduction in the standard deviation of bead coverage. As shown in Figure 8a,b, the optimal uniformity of the bead distribution is observed at 18.46 µL·s^−1^, where bead coverage is high and its standard deviation minimized. While a flow velocity of 15.54 µL·s^−1^ yields high bead coverage, the concomitant concentration of beads in the first aggregation zone elevates the coverage standard deviation and compromises distribution uniformity. The axial coverage profile is steadier at a flow velocity of 18.46 µL·s^−1^ (refer to Appendix C), which confirms the improvement of magnetic bead distribution uniformity. The uniformity of magnetic bead distribution is jointly regulated by acceleration time and average flow rate. A lower flow velocity leads to local aggregation, high coverage, and poor uniformity. Conversely, a higher flow velocity can improve uniformity while maintaining coverage by prolonging the acceleration process. According to the experiments, the optimal average flow velocity was set to 18.46 μL·s^−1^, and the acceleration time is 2 s. Under this condition, the magnetic beads in the microchannel maintain a high level of coverage while the standard deviation of coverage is low, indicating better uniformity in the distribution of magnetic beads.

It is necessary to point out that the optimal values (e.g., 18.46 µL·s^−1^ flow velocity) were obtained under fixed experimental conditions to isolate the effects of fluidic parameters on bead distribution uniformity. The proposed approach is inherently platform-based rather than assay-specific. Since the magnetic bead handling, fluidic control, and signal acquisition modules operate independently of the immunochemistry used for target recognition, the optimized parameters can be directly applied to detect different analytes by simply replacing the corresponding antibodies in the immunoassay kits without extra optimization. However, when applied to systems with different physical properties, such as changes in reagent viscosity, magnetic bead concentration, or microchannel geometry, these optimal parameters may vary. Crucially, however, our proposed framework, which combines real-time imaging, quantitative homogeneity analysis, and signal correlation, provides a universally applicable method for optimizing the distribution of magnetic beads in any magnetic bead-based ECL system. Thus, while the numerical values may be system-specific, the approach itself is transferable and offers a practical strategy for enhancing detection performance across different assay configurations and clinical applications.

#### 3.1.3. Impact of Magnetic Bead Uniformity on ECL Signal

**Figure 9:**
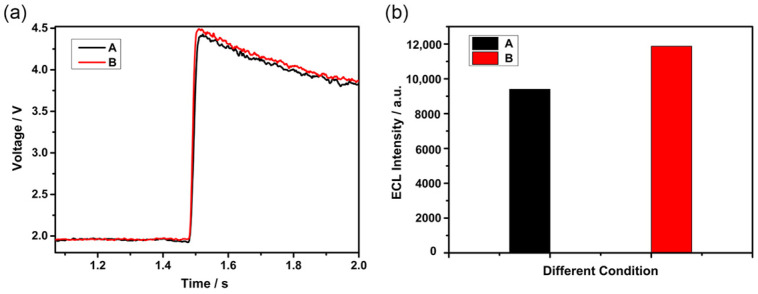
(**a**) V-t response curves for the two experimental conditions (**b**) Calculated photon counts for the two experimental conditions. The symbols A and B in Figures (**a**,**b**) denote two experimental conditions: A (flow velocity: 15.54 µL·s^−1^; acceleration: 1.5 s) and B (flow velocity: 18.46 µL·s^−1^; acceleration: 2 s).

On the basis of Section 3.1.2, this section explores the influence of the uniformity of magnetic bead distribution on the output of ECL signals. According to Figure 8, two conditions with similar coverage but markedly different uniformity of magnetic bead distribution were selected for the following experiment. They are Condition A with a flow velocity of 15.54 µL·s^−1^ and the acceleration time of 1.5 s and Condition B with a flow velocity of 18.46 µL·s^−1^ and the acceleration time of 2 s. The temporal voltage responses and luminescence intensities recorded during the ECL experiment are displayed in Figure 9. As illustrated in Figure 9a, the red curve (Condition B) consistently lies above the black curve (Condition A), demonstrating that at similar levels of bead coverage, greater uniformity in bead distribution on the working electrode surface yields a substantially higher voltage signal amplitude from the reaction. Via antilogarithmic and integration operations, the voltage response curves were further processed, and the corresponding ECL luminescence intensities for the two experimental conditions were yielded, which can be seen in Figure 9b. The enhanced uniformity of bead distribution in Condition B led to a 26.32% increase in ECL luminescence intensity compared to Condition A. This emphasizes the importance of achieving uniform distribution of magnetic beads on the working electrode surface to enhance ECL response and improve overall detection sensitivity.

### 3.2. TSH Assay and Analysis

**Figure 10:**
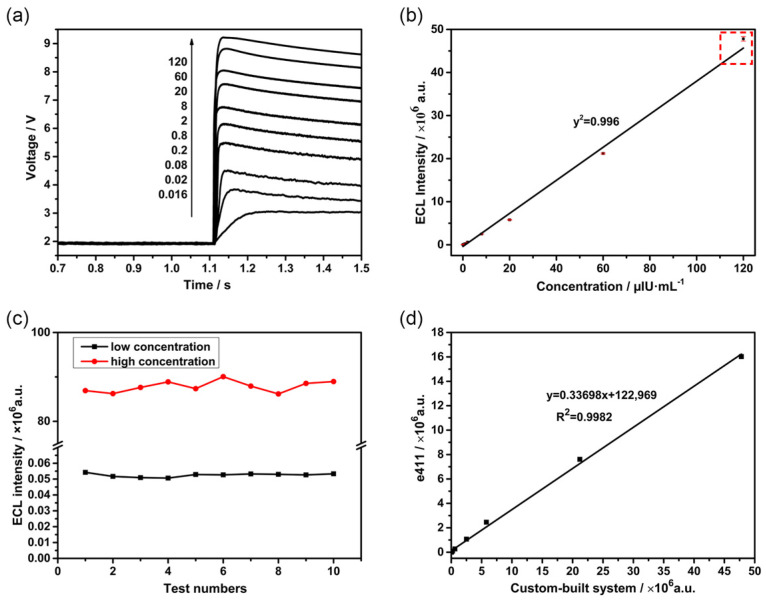
(**a**) V-t curves for different concentration gradients. (**b**) The fitting curve of processed photon counts versus concentration. (The red dashed box marks the data that deviates from the linear dependence at high concentrations. For routine clinical use, samples exceeding 60 µIU·mL^−1^ are recommended to be diluted.) (**c**) Luminescence kinetic curves for high- and low-concentration samples. (**d**) Linear correlation comparison with the Roche Cobas e411 analyzer.

We systematically evaluated the linearity, precision, and accuracy of the ECLIA system to assess its clinical applicability. To begin with, TSH was selected as the target analyte, and the relationship between ECL signal intensity and TSH concentration was tested, as Figure 10b shows. Ten samples with a concentration range of 0.016 to 120 μIU·mL^−1^ were prepared with buffer. Each sample was tested with the aforementioned self-developed system, and raw voltage-time (V-t) curves were recorded, which can be seen in Figure 10a. The results showed a strong linear correlation across the tested range, fitting the equation y = 38,383.2x − 397,430, with a coefficient of determination (R^2^) of 0.996. The system demonstrated a wide linear dynamic range covering four orders of magnitude, with particularly consistent linearity observed at luminescence intensities between 10^3^ and 10^7^.

Subsequently, the precision of the constructed ECLIA system was analyzed and evaluated. Repeatability tests were performed using low- and high-concentration TSH samples within the linear range, with each concentration measured consecutively 10 times [24]. The sample set was carefully selected to ensure adequate representation across clinically relevant categories: low-concentration TSH samples in the hyperthyroid range (<0.4 µIU·mL^−1^), and high-concentration TSH samples in the hypothyroid range (>4 µIU·mL^−1^). The ECL intensities were recorded, and the CV was calculated. As shown in Figure 10c, the CVs for the low- and high-concentration samples were 2.02% and 1.43%, respectively, indicating that the developed ECLIA device exhibits excellent repeatability across both low and high TSH concentrations. As shown in Figure 10b, the highest TSH concentration point (120 µIU·mL^−1^) exhibits a slight positive deviation from the linear regression line (the data marked by the red dashed box). This deviation is attributable to the nonlinear gain characteristics of the PMT under high instantaneous photon flux, rather than nonlinearity in the immunoassay chemistry itself. For routine clinical use, samples exceeding 60 µIU·mL^−1^ are recommended to be diluted to ensure operation within the optimal linear range of the PMT detector. The assay remains linear up to 120 µIU·mL^−1^ (R^2^ = 0.996), and the slight deviation at the upper extreme does not compromise clinical utility, as hyperthyroid patients requiring accurate quantification typically have TSH concentrations below 10 µIU·mL^−1^.

A limitation of the current study is that method comparison was assessed solely through correlation analysis. In future work, we will perform a comprehensive Bland–Altman bias analysis with a larger cohort of clinical samples to further validate the agreement between our system and established reference methods, particularly at the lower end of the clinical decision range where precision is most critical for thyroid function assessment.

To assess the clinical adequacy of the developed ECLIA system for TSH detection, we compared its analytical performance with established clinical decision ranges and guideline recommendations. According to the guidelines released by National Academy of Clinical Biochemistry (NACB), the functional sensitivity (defined as the concentration at which the inter-assay CV ≤ 20%) for a third-generation TSH assay should be ≤0.02 µIU·mL^−1^ [25]. Our system achieved a lower limit of quantitation (LLOQ) of 0.016 µIU·mL^−1^ with a CV of 2.02% at low concentration, which not only meets but substantially exceeds the third-generation assay requirement. The linear range of 0.016–120 µIU·mL^−1^ encompasses the entire clinical reporting range, including the hyperthyroid threshold (<0.02 µIU·mL^−1^), euthyroid reference interval (0.4–4 µIU·mL^−1^), and hypothyroid concentrations (>4 µIU·mL^−1^), without requiring sample dilution. These comparisons demonstrate that the analytical performance of our system is fully compatible with clinical diagnostic requirements for thyroid function assessment.

Finally, the accuracy of the constructed ECLIA system was assessed. In Figure 10d, the TSH detection results obtained with the custom-built system were compared with those from the Roche ECLIA analyzer. A rigorous correlation analysis confirmed high comparability between the results of the in-house instrument and the commercial Roche Cobas e411 system. The measurement accuracy of the aforementioned self-developed system was proven to be comparable to the standard reference method throughout the entire testing range with a high correlation coefficient (R^2^ > 0.998). In addition, the linear relationship indicates that the ECL signals generated by the self-developed system were significantly higher than those from the commercial reference system (Roche Cobas e411), which yields a higher signal-to-noise ratio.

## 4. Conclusions

In this work, an integrated electrochemiluminescence immunoassay platform was developed to systematically investigate the effect of magnetic bead distribution on ECL signal performance. A quantitative evaluation method based on image acquisition and analysis was established to characterize bead distribution uniformity on the working electrode surface. By optimizing fluidic parameters, particularly flow velocity, the uniformity of magnetic bead deposition was significantly improved, resulting in enhanced luminescence intensity. This study demonstrates that magnetic bead distribution uniformity is a critical factor influencing ECL signal efficiency and detection reliability. The proposed quantitative framework provides a practical approach for evaluating and optimizing bead deposition processes in ECLIA systems. Analytical validation using TSH standards showed a wide linear detection range, high repeatability, and good agreement with a commercial instrument, confirming the feasibility of the proposed strategy. Rather than introducing new assay chemistry, this work establishes a general methodology for improving signal performance through distribution optimization, providing useful guidance for the design of high-precision bead-based ECL detection systems.

## Figures and Tables

**Figure A1:**
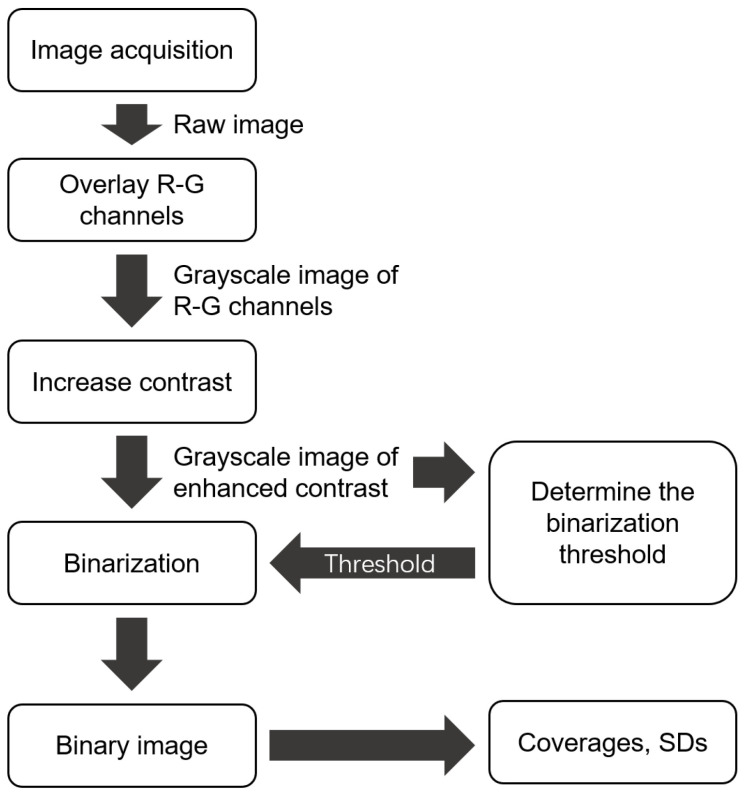
Image processing flowchart.

**Figure A2:**
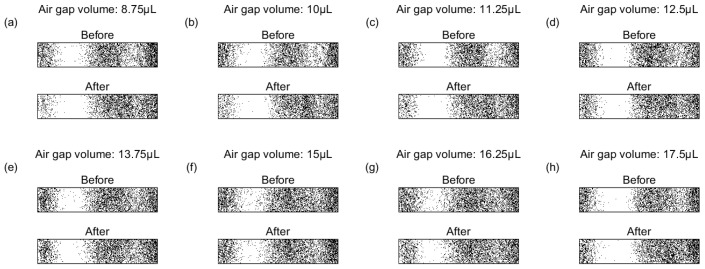
Binary images of magnetic beads distribution captured both before and after the expulsion of the air gap. White pixels indicate magnetic bead coverage, and black pixels indicate the exposed working electrode. Figures (**a**–**h**) show a sequential increase in the air gap volume.

**Figure A3:**
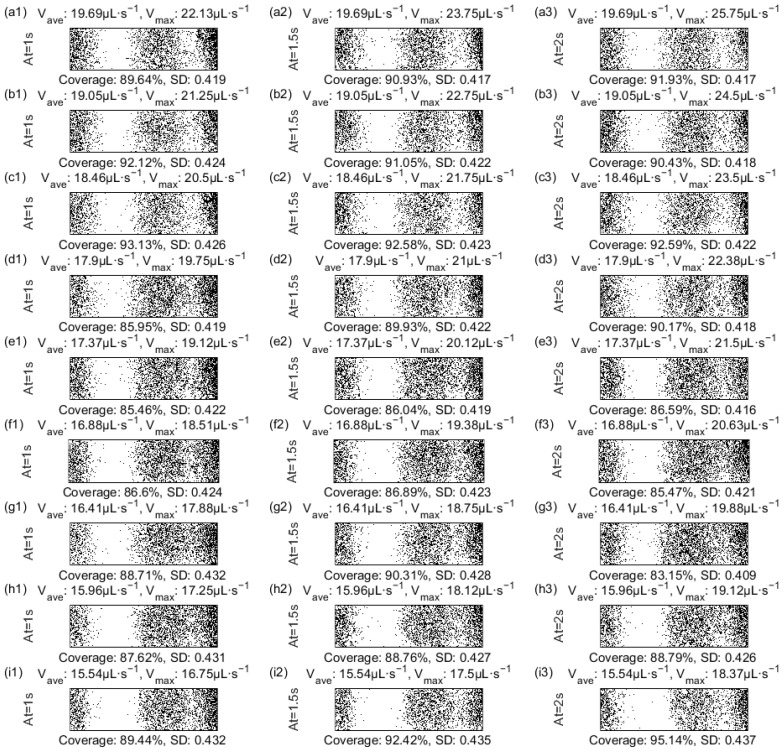
(**a1**–**i3**) Binary images of magnetic beads distribution captured varying the flow velocities. White pixels indicate magnetic bead coverage, and black pixels indicate the exposed working electrode. Each subfigure is annotated below with the corresponding bead coverage and coverage SD. A constant acceleration time is maintained for all images in a given column (e.g., (**a1**) to (**i1**)), corresponding to a decrease in average velocity from top to bottom. Conversely, a fixed average velocity is maintained for all images in a given row (e.g., (**a1**) to (**a3**)), with acceleration increasing from left to right.

## Data Availability

Data are contained within the article.

## Abbreviations

The following abbreviations are used in this manuscript:

ECLIA	Electrochemiluminescence immunoassay
ECL	Electrochemiluminescence
TPrA	Tripropylamine
POCT	Point-of-care testing
TSH	Thyroid-stimulating hormone
PMT	Photomultiplier tube
PEEK	Polyetheretherketone
V-t	Voltage-time
SD	Standard deviation
R-G	Red and green
PBS	Phosphate-buffered saline
CV	Coefficient of variation

## Appendix A

The image processing workflow for quantitative analysis of magnetic bead distribution on the working electrode surface consists of the following sequential steps:

Step 1: Image Acquisition. Raw images of the magnetic bead-covered electrode surface within the microchannel are captured using a high-resolution camera (2048 × 2048 pixels) under consistent LED illumination in a dark room environment.

Step 2: Channel Extraction and Overlay. The red (R) and green (G) channels are extracted from the raw RGB images and overlaid. This step enhances the contrast between beads and background by leveraging the distinct color characteristics of the beads.

Step 3: Grayscale Conversion. The overlaid R-G images are converted to a grayscale image.

Step 4: Contrast Enhancement. The grayscale images undergo contrast enhancement to amplify the intensity difference between bead-covered regions and bare electrode areas, improving the discrimination capability for subsequent segmentation.

Step 5: Threshold Determination. The binarization threshold is established by analyzing the grayscale value distribution. Through random sampling of numerous grayscale images from the microchannel and labeling pixels as either magnetic beads or exposed electrode surface, the characteristic grayscale ranges for pixels from magnetic beads or the working electrode are determined, enabling selection of an optimal threshold value.

Step 6: Image Binarization. Using the threshold determined in the previous step, the enhanced grayscale images are segmented into binary format. White pixels represent areas covered by magnetic beads, while black pixels correspond to the bare working electrode substrate.

Step 7: Quantitative Analysis. From the binary images, the bead coverages and the coverage SDs can be calculated.

## Appendix B

Binary images of the distribution of magnetic beads on the working electrode surface in the central area of the microchannel varying the air gap volume, captured both before and after the expulsion of the air gap. White pixels denote areas occupied by magnetic beads, whereas black pixels indicate the bare electrode surface devoid of beads. Comparison of the paired images shows that the least effect was seen with a 15 μL air gap.

## Appendix C

Binary images of the distribution of magnetic beads on the working electrode surface in the central area of the microchannel varying the flow velocities. Each subfigure is labeled with the flow velocity (the average velocity denoted by V_ave_ and the max velocity denoted by V_max_) in its title, acceleration time (denoted by At) in its y-label, and the resulting bead coverage and coverage standard deviation provided beneath it.
